# Is melatonin associated with pro-inflammatory cytokine activity and liver fibrosis in non-alcoholic fatty liver disease (NAFLD) patients? 

**Published:** 2021

**Authors:** Masoudreza Sohrabi, Ali Gholami, Bahareh Amirkalali, Mahsa Taherizadeh, Mahsa Kolahdoz, Fahimeh SafarnezhadTameshkel, Sheida Aghili, Marzieh Hajibaba, Farhad Zamani, Mohsen Nasiri Toosi, Hossein Keyvani

**Affiliations:** 1 *GastroIntestinal and liver Diseases Research Center (GILDRC) , Iran University of Medical sciences, Tehran, Iran *; 2 *Noncommunicable Diseases Research Center, Neyshabur University of Medical Sciences, Neyshabur, Iran *; 3 *Department of Epidemiology & Biostatistics, School of Public Health, Neyshabur University of Medical Sciences, Neyshabur, Iran*; 4 *Iranian Institute Pasture, Tehran, Iran *

**Keywords:** Fatty liver, Cytokine, Melatonin, Inflammation

## Abstract

**Aim:**

The associations between serum levels of melatonin and concentrations of tumor necrosis factor (TNF)-a and interleukin (IL)-6 were assessed among patients with different degrees of non-alcoholic fatty liver disease.

**Background:**

Non-alcoholic fatty liver disease (NAFLD) has become a very common worldwide disease.

**Methods:**

In this cross-sectional study, adult patients diagnosed with fatty liver disease by Fibroscan evaluation were included if they met the inclusion/exclusion criteria for NAFLD. The participants were categorized into the three following groups: 1) fibrosis> 9.1KP and steatosis >290 dbm; 2) fibrosis: 6-9.0 KP and steatosis 240-285; and 3) fibrosis < 5.8 KP and steatosis<240 dbm. Post-fasting, 5 ml of venous blood was collected for laboratory assessment, and a questionnaire including demographic, anthropometric, laboratories and clinical data was completed.

**Results:**

A total of 97 participants were included. The mean age was 42.21±11 years, and 59 patients (60.0%) were female. Melatonin levels as well as pro-inflammatory cytokines levels were correlated with advancing fibrosis and steatosis in univariate analysis. A significant association was observed between these cytokines and advancing fibrosis, severe steatosis levels, and melatonin concentrations. Furthermore, in the multiple linear regression model, melatonin levels showed a significant association with these cytokines.

**Conclusion:**

Melatonin may have protective effects on tissue injury during advancing liver fibrosis via cytokines modulation. Therefore, it can be considered as a potential therapeutic management strategy for NAFLD.

## Introduction

 Non-alcoholic fatty liver disease (NAFLD) has become a very common worldwide disease. It is caused mainly by the over-accumulation of fat in hepatocytes in the absence of alcohol consumption and/or other liver diseases. It has become an increasingly important health concern because of its morbidity and associations with metabolic syndrome factors. Furthermore, this condition has a wide array of clinical presentations, from simple steatosis to severe fibrosis, with a consequent risk of hepatocellular carcinoma. This condition can cover a broad spectrum of abnormalities as seen in laboratory analyses (1, 2).

The pathophysiology of NAFLD development, progress, and its consequences remain unclear. Insulin resistance (IR), immunity, and oxidative stress are considered pathophysiological mechanisms within NAFLD (3). It is hypothesized that NAFLD progression may occur as a result of the interaction between different factors, including environmental as well as genetic susceptibilities, and the immune system (4, 5). In this regard, multiple-hit hypotheses were introduced (6). In these contexts, insulin resistance, hormones secreted from adipose tissue, nutritional factors, gut microbiota, and genetic and epigenetic factors were considered in the development of NAFLD. This research suggests that inflammatory pathways may require serious attention. It is assumed that insulin resistance (IR) plays a major role in the development of liver steatosis (3). Previous studies have shown that the innate immune system and cytokines, as major mediators, have vital roles in controlling the inflammation process that has been observed in NAFLD. In addition, IR (as a main factor in NAFLD development) is often associated with chronic inflammation (7). IR also promotes immune system reactivation and the release of pro-inflammatory cytokines (8). In addition, adipose tissue inflammation is characterized by an increased expression of various pro-inflammatory cytokines (9) compared to normal inflammatory states. Cytokines are able to mitigate many key features of liver diseases, including acute liver failure, acute phase response, steatosis, cholestasis, hypergammaglobulinemia, and fibrosis development (10).

The main pro-inflammatory cytokines are tumor necrosis factor-alpha (TNF-α) and IL-6 (11). TNF-α is involved in the pathophysiology of various aspects of NAFLD. TNF-α expression is increased in obese patients, and its levels decrease following weight loss (9, 12). Interleukin-6 (IL-6) is a pro-inflammatory cytokine that is secreted mainly from T lymphocytes, and it basically activates monocytes and macrophages while also stimulating B lymphocyte differentiation and antibody secretion (13). Both TNF-α and IL-6 have been associated with obesity and insulin resistance and can alter insulin sensitivity by prompting different phases in the insulin signaling pathway, stimulating hepatic lipogenesis, and impairing insulin signaling (14, 15).

Melatonin, however, is a pineal secretory product involved in the protection of cell injuries (due to a variety of toxic oxygen- and nitrogen-based reactants) and in the stimulation of antioxidative enzymes. Melatonin is also secreted from other organs such as the gastrointestinal system, the skin, and thrombocytes and lymphocytes (16). Melatonin (MT) demonstrates an antioxidant effect in protecting cells and tissues from radical damage, while also inhibiting pro-inflammatory cytokines including TNF‐α, IL‐1β, and IL‐6 b during the development of hepatic fibrosis. All together melatonin has regulatory effects on immunity and anti-inflammation pathways(17, 18). Animal as well as human studies have revealed the positive effects of melatonin as a medication on multiple different disorders, including liver injuries (13, 19). 

Melatonin has been seen to reduce hepatocyte damage and inflammation by preventing pro-inflammatory cytokine production and NOD-like receptor pyrin domain (containing 3 (NLRP3) inflammatory actions), decreasing oxidative stress, and reducing hepatocyte death (20, 21). Despite melatonin’s effects, its known interactions with pro-inflammatory substances in the liver are controversial, particularly in NAFLD. The present study aimed to explore associations between melatonin and pro-inflammatory cytokines according to different stages of liver fibrosis. 

## Methods

**Study Population**

This cross-sectional study was carried out among patients referred to the liver clinic at Firoozgar Hospital between June 2017 and January 2019. The inclusion criteria were being an adult and the presence of liver steatosis shown in transabdominal ultrasonography. Exclusion criteria were the presence of viral hepatitis; auto-immune hepatitis; hepatic metabolic diseases; post-treatment for HCV infection; diabetes mellitus; bariatric surgery; taking medication which affects liver status or melatonin levels (such as selemarin or oral antidiabetic drugs); and alcohol consumption (>30g/day in men and >20g/day in women). All participants underwent Fibroscan (502 touch, Echosense, France). NAFLD diagnosis was confirmed by a consultant gastroenterologist.

Three groups were defined: 1) Normal or control group with normal-range serum liver enzymes along with normal fibrosis (<5.9KP) and steatosis (<240 dbm); 2) Mild to moderate fibrosis (fibrosis between 6-9.0KP and steatosis less than 285 dbm); 3) Severe fibrosis and suspected steatohepatitis (NASH) (elevated liver enzymes, steatosis (>290 dbm) and fibrosis >9.1KP). Patients were matched by age and gender (22).

First, 5 ml of fasting venous blood was taken from each patient for laboratory assessment, which measured serum levels of ALT, AST, triglycerides, total cholesterol, and melatonin. A questionnaire was completed regarding demographic data (age, gender), and anthropometric data (high, weight, waist circumference, hip circumference) was taken. 

**Ultrasonography**

Fatty liver was defined by ultrasonography as normal, mild, moderate, or severe. Normal liver was defined when the liver consistency was homogeneous, displayed fine-level echoes, was minimally hyperechoic or even isoechoic in contrast to a regular renal cortex. Mild steatosis was defined as a minor increase in liver echogenicity. Moderate steatosis was defined as the existence of increased liver organ echogenicity. Finally, severe steatosis was considered as a marked increase in hepatic echogenicity, poor penetration of posterior segment from the right lobe of the liver, and poor or no visual images from the hepatic vessels and diaphragm (23).

**Liver Transient Elastography**

Transient elastography was performed to detect liver fibrosis and steatosis. This process was carried out by a relevant medical expert in Firoozgar Hospital using Fibroscan (Fibroscan; Echosense, Paris, France). The examination was performed according to standard protocol, laying the patient in the dorsal decubitus position with maximum right arm abduction. For each patient, at least ten successful shots were considered as a complete exam. The results of fibrosis were reported in kilopascals (kPa). Following the manufacturer’s guidelines, the median value of successful measurements was considered as overall liver stiffness. The control attenuated parameter (CAP) is a device that can evaluate the steatosis status of the liver during Fibroscan evaluation with both M and XL probes. According to previous studies of liver biopsies, the CAP value represents the grade of steatosis (22).

**Ethical Considerations**

This study was approved by the Ethics Committee of Iran University of Medical Sciences, according to the Helsinki Declaration (ethical code IR.IUMS1397.32992). Written informed consent was obtained from each patient before enrollment.

**Laboratory Methods**

The blood samples were transferred to a central lab; then plasma and peripheral blood mononuclear cells (PBMC) were separated. After separating the buffer coat, PBMC was isolated using Phosphate Buffered Saline (PBS) as a buffer and then adding ficoll. Fasting blood sugar (FBS), aspartate aminotransferase (AST), alanine transaminase (ALT), IL-6, TNF-α, and melatonin were measured with ELISA kits (Quiagen, Germany).

**Data Analysis**

Descriptive data is presented as frequencies, percentages, and mean ± SD. The univariate linear regression model was used to investigate associations between dependent variables (IL-6 and TNF-α) and melatonin and other variables. Multiple linear regression models (with the backward method) were used to assess the independent effect of melatonin on IL-6 and TNF. Data was analyzed by SPSS software (IBM-SPSS, version 20.0 IL, USA). The significance level of the analyses was *p* < 0.05.

## Results

In total, 97 subjects were enrolled in the study. Table 1 illustrates the basic patient characteristics. Patients were allocated into three groups according to Fibroscan results. Mean patient age was 42.2±11.3 years, and59 (60%) patients were female. In the descriptive analysis, it was observed that age (*p*<0.001), gender (*p*<0.02), and BMI (*p*<0.001) were associated with fibrosis in groups 1 through 3 (Table 1). Similar results were observed with regard to fasting blood sugar (FBS), total cholesterol, triglycerides, alanine transferase (ALT), and aspartate transaminase (AST). Furthermore, serum levels of melatonin, IL-6, and TNF-α also showed an increasing trend from groups 1 to 3. 

In the univariate analyses, it was observed that increasing IL-6 and TNF-α levels were significantly associated with liver enzymes according to the three groups, with increasing levels of melatonin, and with steatosis and fibrosis (Tables 2-4). In the multiple linear regression analyses (Table 4), it was further observed that IL-6 and TNF-α levels were positively related to melatonin levels (*p *< 0.001, 95% CI = 0.13-0.24 and *p*= 0.003, 95% CI = 0.07-0.31, respectively).

**Table 1:** Demographic and laboratory findings of patients with different stages of fibrosis

Variable	Total (N=97)	Normal (N=42)	Mild to moderate (N=28)	Severe (N=27)	*p*-value
Age (year)	42.2 ±11.3^*^	36.5 ±10.1	45.1 ± 11.4	47.4± 10.0	<0.001
Gender					
Female	59	23	23	13	0.02
Male	38	19	5	14	
BMI					
<25	28	23	4	1	<0.001
≥25	69	19	24	26	
Total cholesterol (mg/dl)	189.6±52.2	169.0 ±45.3	210.4 ± 64.1	205.4 ± 36.1	0.02
LDL (mg/dl)	120.2 ±33.5	114.7 ± 31.2	130.0 ± 42.3	120.1 ±25.2	0.24
HDL (mg/dl)	43.8±8.9	44.2± 9.3	44.8 ± 9.4	41.9 ± 7.7	0.55
Triglycerides (mg/dl)	161.1±89.8	124.1± 60.0	189.4 ± 97.2	197.0 ± 103.4	0.001
AST (U/L)	40.7±31.1	21.4±7.3	53.6 ±37.7	57.4 ±30.7	<0.001
ALT (U/L)	48.8±41.3	21.0± 10.7	66.1 ±46.2	74.0 ±40.7	<0.001
**Alkaline phosphatase (U/L)**	193.2±61.8	159.9± 53.8	215.5± 64.2	221.9± 45.6	<0.001
FBS	107.9±34.9	92.8 ± 10.8	110.3 ± 23.9	137.7± 57.7	<0.001
Melatonin	457.1±496.1	332.4±459.8	494.4±507.0	610.3±508.0	0.07
IL-6 (pg/ml)	125.7±115.3	91.0±97.4	145.8±120.5	158.9±124.5	0.03
TNF-α (pg/ml)	202.0±199.3	115.6±153.1	257.8±202.2	278.5±214.1	<0.001

^* ^mean ± SD; LDL: Low-density lipoprotein; HDL: High-density lipoprotein; AST: Aspartate transaminase; ALT: alanine aminotransferase; FBS: Fasting blood sugar

**Table 2.** Univariate linear regression analyses of melatonin association with IL-6

Variables	Coefficient	Std. Err	t	*p*-value	95% CI
Gender (Female)	-21.9	24.0	-0.91	0.364	-69.55, 25.72
Age	0.99	1.04	0.95	0.345	-1.08, 3.05
BMI	0.50	0.32	1.55	0.124	-0.14, 1.13
Total cholesterol (mg/dl)	0.29	0.24	1.18	0.240	-0.20, 0.78
LDL (mg/dl)	-0.15	0.40	-0.38	0.71	-0.94, 0.64
HDL (mg/dl)	1.12	1.47	0.77	0.446	-1.80, 4.06
Triglycerides (mg/dl)	0.30	0.14	2.10	0.039	0.02, 0.58
AST (U/L)	1.30	0.36	3.66	<0.001	0.60, 2.10
ALT (U/L)	0.95	0.27	3.52	0.001	0.41, 1.48
Alkaline phosphatase (U/L)	0.34	0.19	1.79	0.079	-0.04, 0.71
FBS	0.13	0.38	0.33	0.742	-0.63, 0.89
Stages of fibrosis					
Mild to moderate	54.77	27.38	2.00	0.048	0.41, 109.15
Severe	67.90	27.38	2.45	0.016	12.94, 122.87
Steatosis					
Mild-Moderate	-11.93	31.73	-0.38	0.708	-74.94, 51.08
Severe	65.62	26.15	2.51	0.014	13.70, 117.55
					
Melatonin	0.18	0.02	11.54	<0.001	0.15, 0.21

LDL: Low-density lipoprotein; HDL: High-density lipoprotein; AST: Aspartate transaminase; ALT: alanine aminotransferase; FBS: Fasting blood sugar

**Table 3:** Univariate linear regression analyses of melatonin association with TNF-α

Variables	Coefficient	Std. Err	t	*p*-value	95% CI
Gender (Female)	-11.56	41.67	-0.28	0.782	-94.28, 71.16
Age	2.83	1.80	1.57	0.119	-0.75, 6.42
BMI	1.10	0.55	2.01	0.047	0.01, 2.18
Total cholesterol (mg/dl)	0.50	0.43	1.15	0.252	-0.36, 1.35
LDL (mg/dl)	0.02	0.70	0.02	0.982	-1.37, 1.40
HDL (mg/dl)	1.36	2.60	0.52	0.602	-3.81, 6.53
Triglycerides (mg/dl)	0.41	0.24	1.67	0.100	-0.08, 0.90
AST (U/L)	2.52	0.60	4.17	<0.001	1.32, 3.72
ALT (U/L)	2.01	0.45	4.45	<0.001	1.32, 3.72
Alkaline phosphatase (U/L)	0.71	0.32	2.20	0.030	0.70, 1.35
FBS	0.68	0.68	0.99	0.325	-0.69, 2.05
Stages of fibrosis					
Mild to moderate	142.15	45.41	3.13	0.002	51.99, 232.31
Severe	162.88	45.91	3.55	0.001	71.73, 254.05
Steatosis					
Mild-Moderate	-13.80	53.99	-0.26	0.799	-121.00, 93.40
Severe	134.27	44.49	-0.26	0.799	45.93, 222.62
Melatonin	0.28	0.03	9.17	<0.001	0.22, 0.34

LDL: Low-density lipoprotein; HDL: High-density lipoprotein; AST: Aspartate transaminase; ALT: alanine aminotransferase; FBS: Fasting blood sugar

**Table 4:** Multiple linear regression analyses of melatonin association with IL-6 (pg/ml) and TNF-α (pg/ml)

	Variables	Coefficient	Std. Err	t	*p*-value	95% CI
IL-6	Melatonin	0.18	0.03	6.84	<0.001	0.13, 0.24
TNF-α	Melatonin	0.19	0.06	3.19	0.003	0.07, 0.31

## Discussion

NAFLD is increasingly becoming a common health problem worldwide, ranging from simple steatosis to steatohepatitis and advanced fibrosis. The results of the present study revealed that melatonin, IL-6, and TNF-α levels have a significant increasing trend with the progression of liver fibrosis. 

Increasing levels of TNF-α were observed as a pro-inflammatory cytokine. This cytokine is produced by T and B lymphocytes that likely play vital roles in the progression of fatty liver disease (14, 24). In NASH, this cytokine has a lipogenic and fibrogenic effect that eventually induces liver fibrosis (25, 26). Previous studies have shown that TNF-α levels are increased in NASH subjects compared to controls and also that TNF-α levels are correlated with HOMA-IR and NAFLD severity (14, 27).Consistent with the present findings, Plessis et al. revealed that TNF-α levels increased in patients with NASH and were associated with liver enzymes (28, 29). Moreover, the present study observed a correlation between levels of IL-6 in patients and fibrosis and liver enzymes. IL-6 is a multifunctional cytokine that regulates immune responses, acute phase reactions, and hematopoiesis, and it may play a central role in processes from inflammation to tissue injury. This cytokine, as with TNF-α, induces hepatic lipogenesis and IR, impairs insulin signaling, and can modify insulin sensitivity. The underlying mechanism of the effect of IL-6 on steatosis is not yet fully clear (30, 31). In their animal study, Vida et al. showed that the chronic replacement of IL-6 with physiological doses in IL-6-/- mice seriously exacerbated the steatosis induced by a high-fat diet (31). In the present study, the increasing of these two cytokines was associated with steatosis and fibrosis, but this may be due to their ability to work on mitochondria function and oxidation (32, 33). Notably, these cytokines were correlated with transaminase levels and with the severity of the steatohepatitis, suggesting that they may be important mediators in NAFLD-to-NASH progression. 

The present study revealed that the increasing levels of melatonin were associated with pro-inflammatory cytokines concentrations (Table 4). The effect of melatonin on the level of pro-inflammatory cytokines is not fully understood, particularly in NAFLD patients. Regarding the findings of previous studies, treatment with high doses of melatonin may induce the inhibitory effects on pro-inflammatory cytokines. The majority of these studies focused on animals in which liver diseases had been induced (34, 35). However, there is a lack of data about association melatonin and pro-inflammatory levels in humans with NAFLD (according to levels of liver damage). Although In present study the cirrhotic patients were not enrolled, it has been demonstrated that melatonin is significantly elevated in patients with advanced fibrosis and cirrhosis, particularly in the daytime. This fact may be related to melatonin metabolism disturbances (36, 37). Furthermore, alongside these findings the authors suggest that the high levels of melatonin and cytokines seen in the present study may indicate the need for higher melatonin levels to have any effect on modulating cytokine activity; other pathways should also be considered in terms of developing or inhibiting inflammation and melatonin levels. In addition, patients with NAFLD may suffer from other abnormalities that influence the concentrations and effectiveness of melatonin as well as other pro-Inflammatory and inflammatory substances.

From a clinical point of view, Pakravan et al. demonstrated in clinical trials that the administration of melatonin may be helpful as a treatment for NAFLD (34). Furthermore, some researchers have found that melatonin supplementation was able to improve hepatic mitochondrial function, reducing oxidative stress and increasing the activities of complexes I and IV of the mitochondrial respiratory chain (38). Das et al. found that melatonin played a protective role on mitochondrial dysfunction and that it was able to de-activate liver fibrogenesis (39). In addition, several studies have shown that melatonin protected against fatty liver mainly by preventing oxidative stress. Evidence has shown that melatonin worked as a regulator of miR-34a-5p and could block NAFLD progression in mice (40). Furthermore, it has been reported that melatonin may have a negative influence on the production of certain cytokines in macrophages by suppressing the TLR9 pathway (41). Some studies have indicated that melatonin might regulate mitochondrial function in diabetes patients and may improve certain fat metabolism parameters in patients with NAFLD. In this context, while melatonin has anti-inflammatory effects, the results of clinical studies on the effects of melatonin on IL-6 are controversial. Srinivasan et al. (42) reported that melatonin increases IL-2, IL-6, IL-12, and interferon (IFN) gamma levels by stimulating cytokine production. Zhou et al., however, found that melatonin may decrease IL-6 and increase IL-2 production (43). A similar result was reported in the administration of melatonin to arsenic-induced liver cell damage (13). In human trials, Sánchez-López showed that the administration of melatonin for 6 months could reduce serum pro-inflammatory cytokines levels (44). In general, therefore, the associations between IL-6 and melatonin remain unclear. 

In conclusion, melatonin may protect the liver from inflammatory damage, although its exact effect and mechanisms are not clear. The protective role of melatonin may be dose-dependent. It should be mentioned that melatonin acts on different pathways that protect against cell injury and modulate immune system processes. Therefore, more studies are needed to cover different aspects of melatonin activity and its correlations with other factors as well as with clinical presentations.

## Conflict of interests

The authors declare that they have no conflict of interest.
